# Diagnostic Accuracy of Three-dimensional Turbo Field Echo Magnetic Resonance Imaging Sequence in Pediatric Tracheobronchial Anomalies with Congenital Heart Disease

**DOI:** 10.1038/s41598-018-20892-2

**Published:** 2018-02-07

**Authors:** QiaoRu Hou, Wei Gao, YuMin Zhong, AiMin Sun, Qian Wang, LiWei Hu, JingLei Wang

**Affiliations:** 1 Diagnostic imaging Center of Shanghai Children’s Medical Center affiliated with Shanghai Jiao Tong University Medical School, Shanghai, China; 2 Department of Pediatric Cardiology of Shanghai Children’s Medical Center affiliated with Shanghai Jiao Tong University Medical School, Shanghai, China

## Abstract

Tracheobronchial anomalies are common in congenital heart disease (CHD), including tracheobronchial stenosis, tracheal bronchus, cardiac bronchus, and bronchial isomerism, which can cause varying degrees of respiratory illness. It is necessary to assess tracheobronchial anomalies and make a preoperative airway evaluation. Multi-slice computed tomography (MSCT) and cardiac magnetic resonance imaging (MRI) are the most effective noninvasive modalities for the diagnosis of CHD and the associated tracheobronchial anomalies. However, MSCT remains an ionizing procedure despite using low dose protocols. The aim of this study was to evaluate diagnostic accuracy of tracheobronchial anomalies in patients with CHD using three-dimensional turbo field echo(3D-TFE) magnetic resonance imaging sequence for preoperative airway evaluation. The results indicated that 3D-TFE provided better image quality as compared to that of 3D-balanced turbo field echo (3D-bTFE), and it can clearly demonstrated the tracheobronchial tree and tracheobronchial anomalies in CHD. This study confirms the clinical value of 3D-TFE in diagnosing tracheobronchial anomalies and supply helpful tracheobronchial information for preoperative strategies and postoperative follow-up.

## Introduction

Tracheobronchial anomalies including tracheobronchial stenosis, tracheal bronchus, cardiac bronchus, and bronchial isomerism are common in congenital heart disease. Cardiovascular anomaly is the principal extrinsic lesion causing tracheobronchial stenosis, including vascular rings, left pulmonary artery sling, and absent pulmonary valve, etc^1–3^. Tracheobronchial stenosis can cause severe respiratory illness. In our institution, the cardiothoracic surgery service routinely requires imaging for airway anatomy prior to surgery. MSCT and cardiac MRI are the most effective noninvasive modalities for the diagnosis of CHD and the associated tracheobronchial anomalies^4–8^. However, MSCT remains an ionizing procedure despite using low dose protocols^4^. MRI has the advantage of being non-ionizing and providing excellent soft tissue contrast for the diagnosis of CHD and tracheobronchial anomalies. Spin echo (SE) sequence can demonstrate the tracheobronchial tree^5,6^, but it is typically a 2D sequence and therefore difficult to depict the entire tracheobronchial tree optimally. 3D sequences which include 3D-bTFE^5–8^ and 3D-TFE can delineate the entire tracheobronchial tree clearly through post-processing. The aim of this study was to evaluate diagnostic accuracy of tracheobronchial anomalies in patients with CHD using 3D-TFE magnetic resonance imaging sequence for preoperative airway evaluation.

## Results

### Patient information

In 75 patients, 25 patients were diagnosed cyanotic CHD and 50 patients were acyanotic CHD. (details see Supplementary Table S1).

### MRI findings

**Figure 1:**
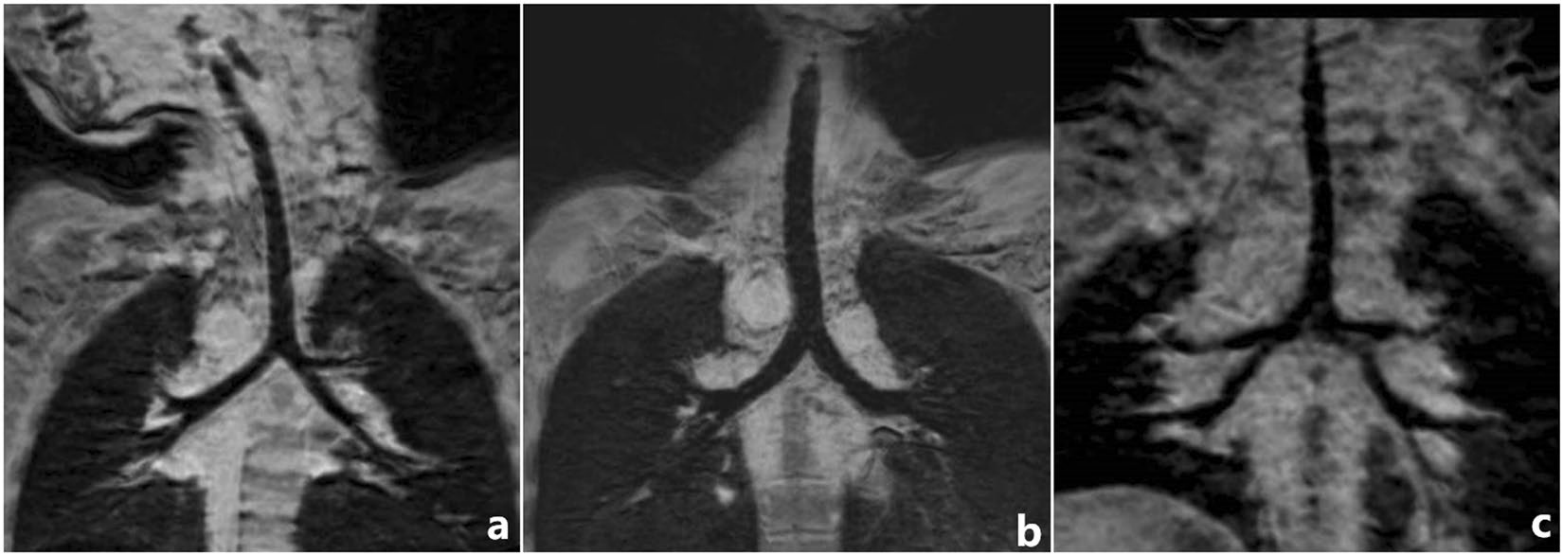
Three-dimensional turbo field echo (3D-TFE) findings of bronchial isomerism and situs inversus types of the tracheobronchial tree. (**a**) Three-year-old girl with situs inversus, single right ventricle, pulmonary atresia and atrial septal defect. 3D-TFE coronal minimum intensity projection (MinIP) reconstruction demonstrated inversus of the right and left bronchi. (**b**) Fifteen-year-old girl with polysplenia syndrome, interrupted inferior vena cava, pulmonary atresia with ventricular septal defect and atrioventricular septal defect, pulmonary stenosis and total anomalous pulmonary venous return. 3D-TFE coronal MinIP reconstruction demonstrated bilateral bilateral left-sided isomerism type of bronchus. (**c**) One-year-old girl with asplenia syndrome, double outlet right ventricle, ventricular septal defect, pulmonary stenosis and total anomalous pulmonary venous return. 3D-TFE coronal MinIP reconstruction demonstrated bilateral right-sided isomerism type of bronchus.

In 75 cases, 49 patients with tracheobronchial anomaly detected by MRI with 3D-TFE as follows: 31 cases had tracheobronchial stenosis, 9 cases had tracheal bronchus, 3 cases had bridging bronchus, 2 cases were situs inversus type of bronchus (Fig. 1a) and 4 cases were isomerism type of bronchus (3 cases were bilateral right-sided isomerism type of bronchus and 1 cases was bilateral left-sided isomerism type of bronchus) (Fig. 1b,c).

**Figure 2:**
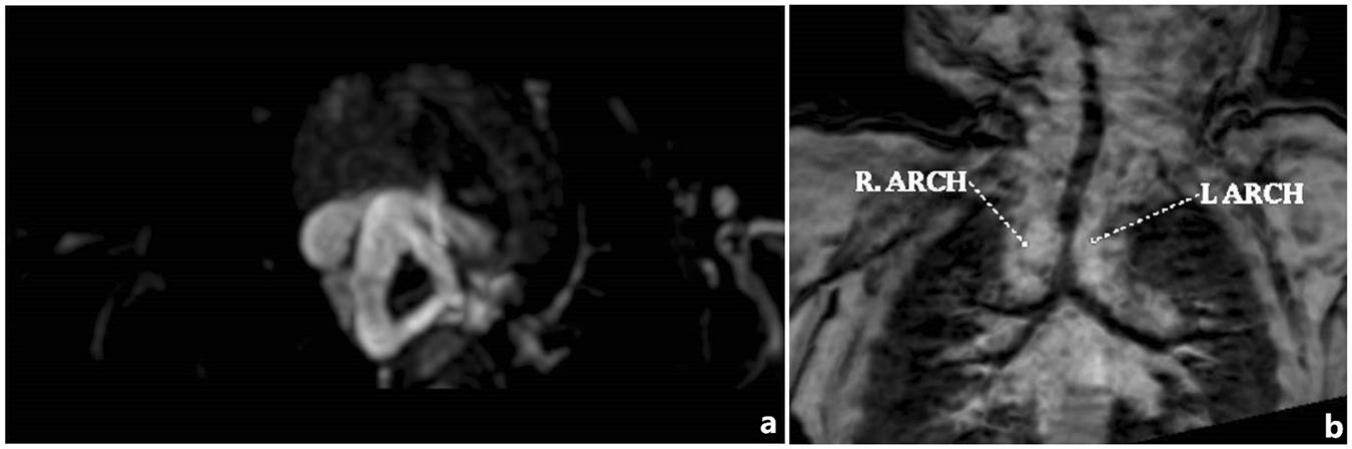
Four-month-old girl with double outlet right ventricle, ventricular septal defect, pulmonary stenosis, atrial septal defect and double aortic arch. (**a**) Contrast-enhanced magnetic resonance angiography axial maximum intensity projection reconstruction demonstrated the double aortic arch and hypoplasia of the left aortic arch. (**b**) Three-dimensional turbo field echo minimum intensity projection reconstruction demonstrated lower segment stenosis of the trachea.

**Figure 3:**
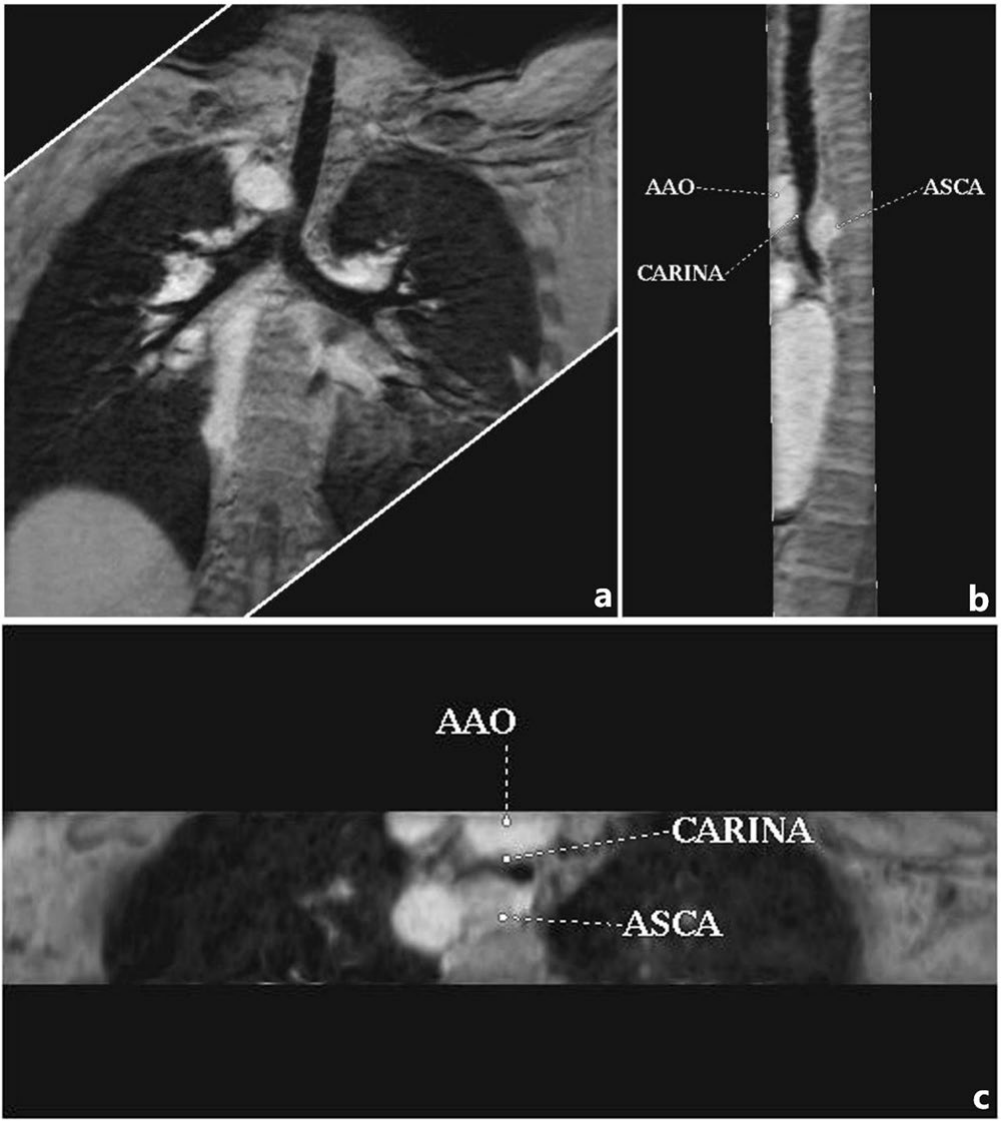
Twelve-year-old girl with corrected transposition of the great arteries, Ebstein’s malformation and right aortic arch with aberrant left subclavian artery. (**a**) Three-dimensional turbo field echo (3D-TFE) coronal MinIP reconstruction demonstrated prominent stenosis of the lower segment of the trachea. (**b**,**c**) 3D-TFE sagittal and axial MinIP reconstruction demonstrated prominent stenosis of the lower segment of the trachea.

**Figure 4:**
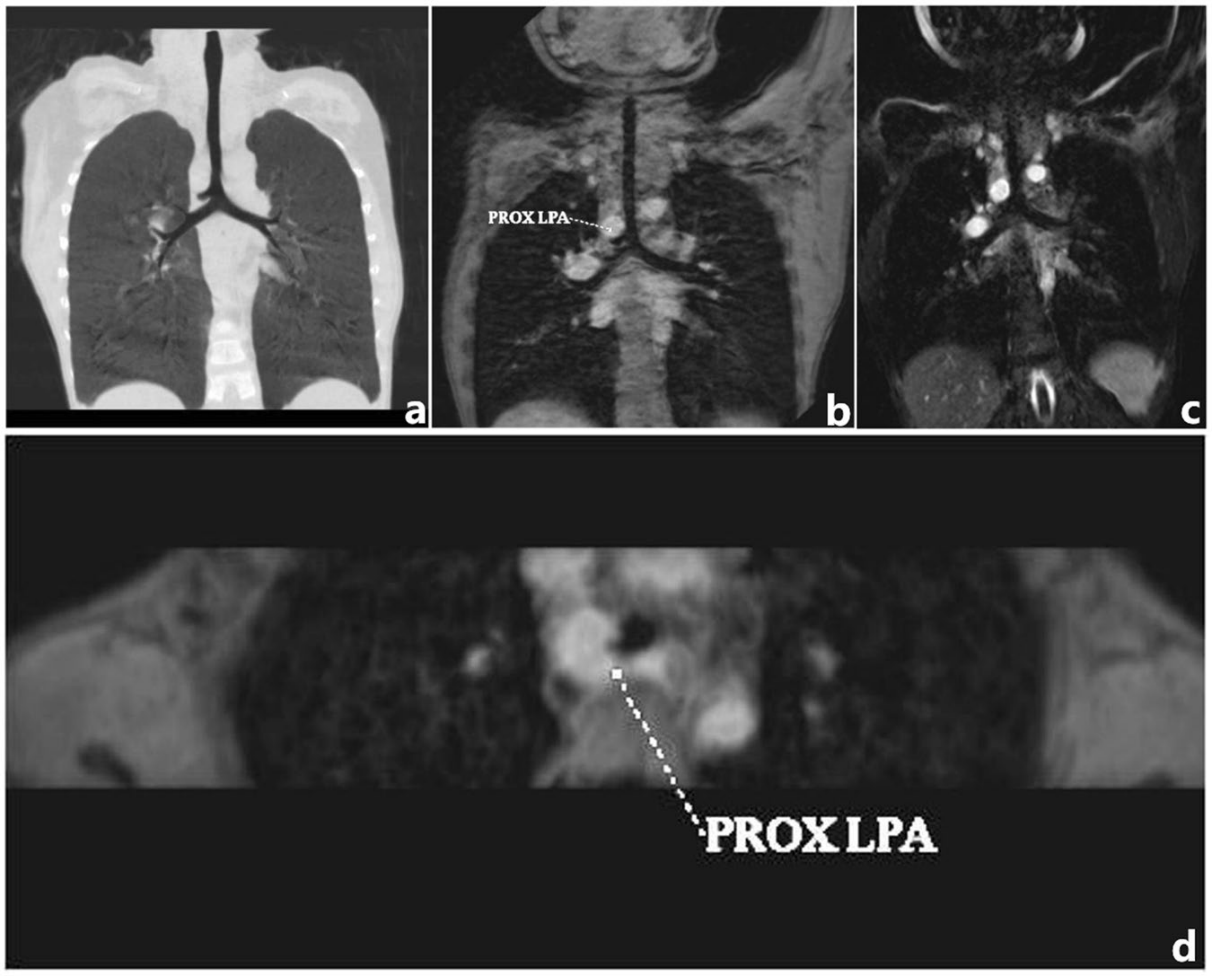
Six-year-old girl with left pulmonary artery sling. (**a**) CT coronal MinIP reconstruction demonstrated stenosis of the mild lower segment of the trachea and tracheal bronchus. (**b**) Three-dimensional turbo field echo (3D-TFE) coronal MinIP reconstruction demonstrated similar tracheobronchial tree findings. (**c**) Three-dimensional balanced TFE coronal MinIP reconstruction demonstrated similar findings, but the margin of the upper trachea was less clear than on 3D-TFE. (**d**) 3D-TFE axial MinIP reconstruction demonstrated that the left pulmonary artery arises from the right pulmonary artery and passes posteriorly between the trachea and esophagus.

**Figure 5:**
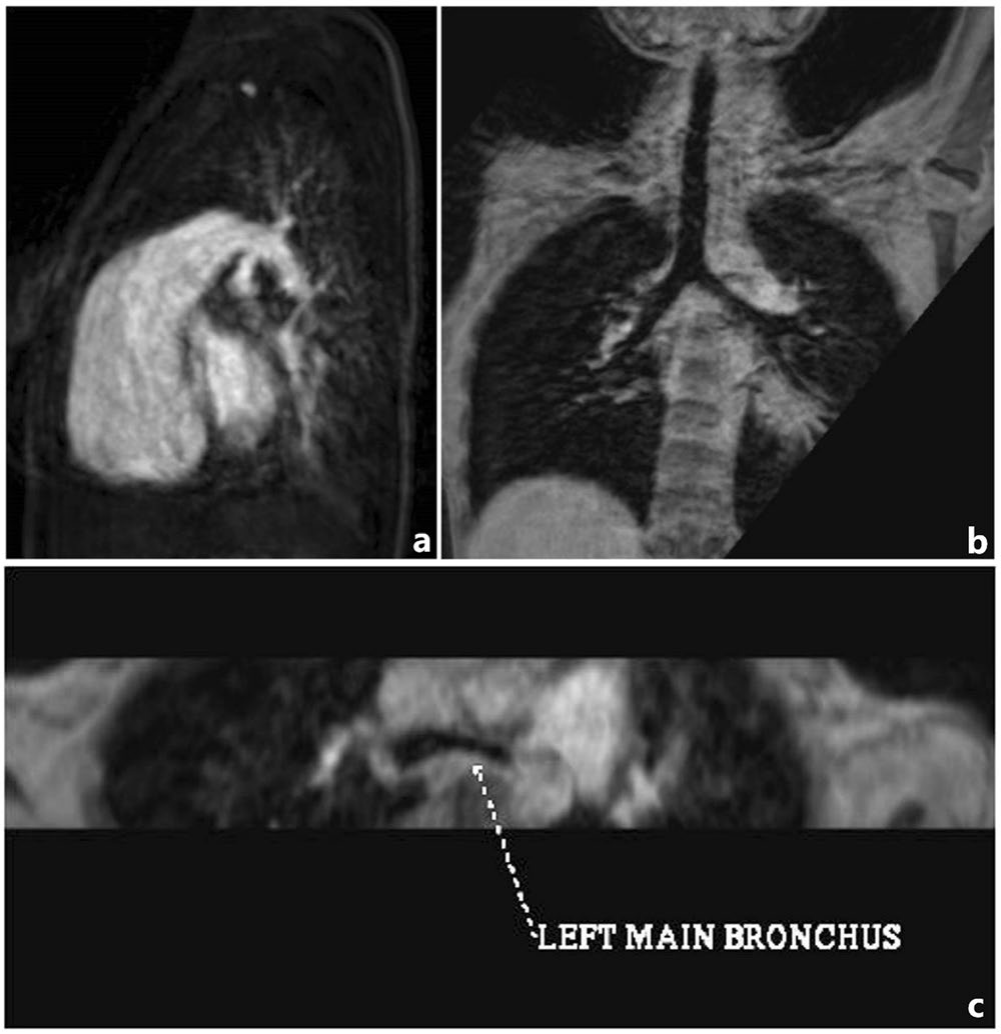
Ten-year-old boy with absent pulmonary valve and intact ventricular septum. (**a**) Contrast-enhanced magnetic resonance angiography axial maximum intensity projection (MIP) reconstruction demonstrated very dilated main pulmonary artery, as expected in this congenital lesion. (**b**,**c**) Three-dimensional turbo field echo coronal and axial MinIP reconstruction demonstrated mild to moderate left bronchial compression by the dilated main pulmonary artery.

**Figure 6:**
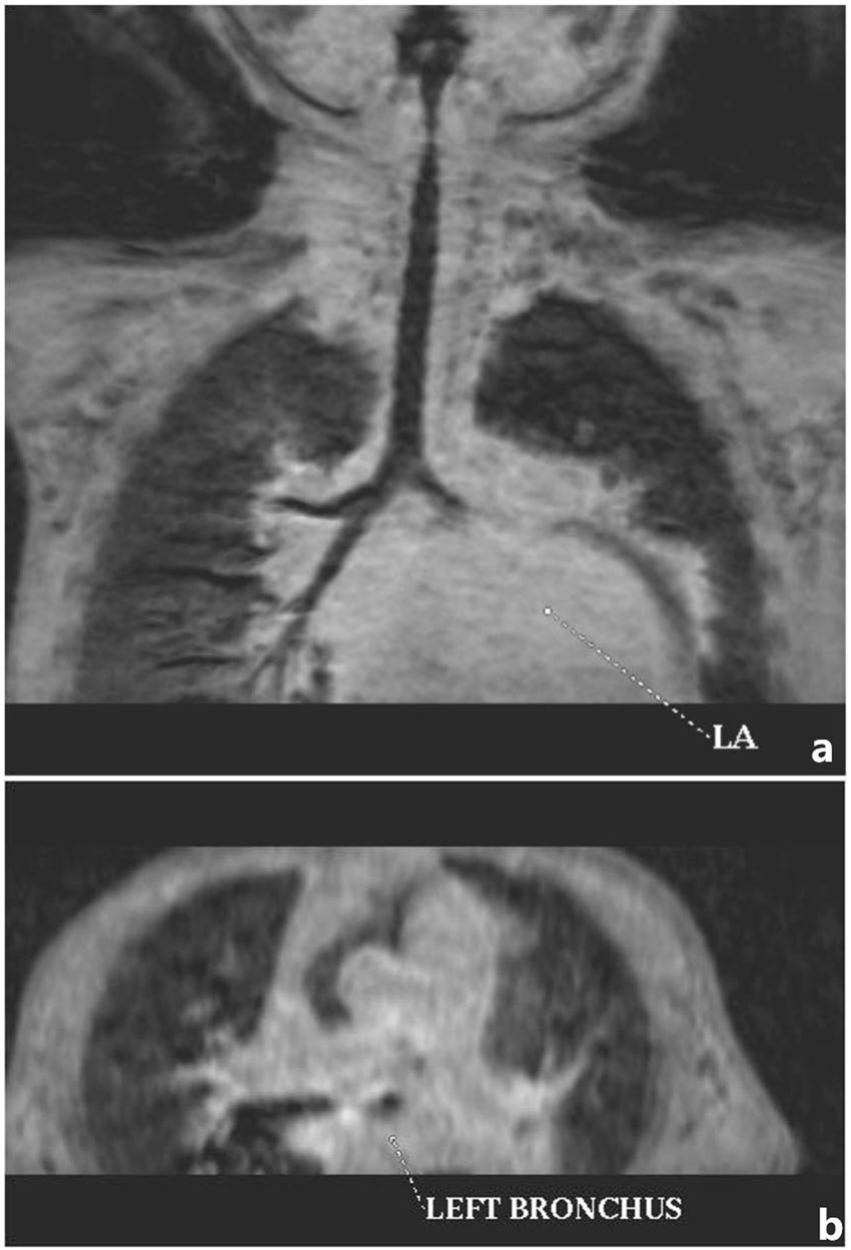
Two-year-old boy with severe mitral valve regurgitation. (**a**,**b**) Three-dimensional turbo field echo coronal and axial minimum intensity projection reconstruction demonstrated severe left bronchial compression by the enlarged left atrium and lower signal intensity in left upper lobe.

In 31 tracheobronchial stenosis cases, the predominant causes of tracheobronchial stenosis were listed in Table 1, such as double aortic arch (Fig. 2), right aortic arch with aberrant left subclavian artery (Fig. 3), left pulmonary artery sling (Fig. 4), absent pulmonary valve (Fig. 5), and enlarged left atrium (Fig. 6).

**Table 1:** The predominant causes of tracheobronchial stenosis.

Cause of tracheobronchial stenosis	Tracheobronchial stenosis
Double aortic arch	3
Right aortic arch with mirror-image branching	0
Right aortic arch + aberrant left subclavian artery + posterior patent ductus arteriosus or ligament	4
left aortic arch + aberrant right subclavian artery	0
Left aortic arch with right descending aorta	2
Innominate artery compression syndrome	1
Left pulmonary artery sling	1
Absent pulmonary artery valve	1
Obvious enlarged left atrium	1
Others	18
Total	31

### Subjective evaluation of image quality

Excellent image quality scores of 3D-TFE (4.61 ± 0.44 and 4.61 ± 0.42) from reviewer 1 and 2 respectively were exhibited in 71 patients. The image quality scores of 3D-bTFE were (3.93 ± 0.54 and 3.95 ± 0.55) and the agreement between the two reviewers was good (*p* values were 0.99 and 0.82 respectively). The difference of the image quality scores between 3D-TFE and 3D-bTFE by the two reviewers were statistically significant (*p* < 0.01).

### Comparing MRI and CT findings

There was good inter-modality agreement, *Kappa 0*.*75*, between 3D-TFE and MSCT for the detection of tracheobronchial anomalies. The validity and efficacy of 3D TFE sequence in the diagnosis of tracheobronchial anomalies were demonstrated in Supplementary Tables S2 and S3.

3D-bTFE sequence was performed in 71 patients (4 patients woke up from sedation and this sequence was not performed) and can demonstrate the tracheobronchial tree but the margin of upper portion trachea was not well visualized due to loss of signal compared with 3D-TFE (Fig. 4b,c). There was also good inter-modality agreement, *Kappa 0*.*62*, between 3D-bTFE and MSCT for the detection of tracheobronchial anomalies (Table 2).

**Table 2:** Validity and efficacy in 3D-TFE and 3D-bTFE sequences.

	3D-TFE	3D-bTFE
sensitivity	91.3%	84.0%
specificity	82.8%	81.0%
accuracy	88.0%	83.1%
positive predictive value (PPV)	89.0%	91.3%
negative predictive value (NPV)	85.7%	65.4%
positive likelihood ratio (PLR)	4.4	4.4
negative likelihood ratio (NLR)	0.1	0.2
*Kappa*	0.75	0.62

## Discussion

Airway disorders are potentially life-threatening, and the interpretation of chest radiographs or images from any other chest imaging modality in the pediatric population is incomplete without airway evaluation^4^. CHD is often associated with tracheobronchial anomalies^1–4^. In our center, the patients who will have corrective surgery for CHD will undergo MSCT or cardiac MRI for preoperative airway evaluation.

MSCT and cardiac MRI are the most effective noninvasive modalities for the diagnosis of CHD with tracheobronchial anomalies^4–9^. MSCT plays an important role in noninvasive imaging of the lungs and airway in pediatric patients due to its higher spatial resolution, greater speed and lower need for sedation^10–12^. However, MSCT utilizes ionizing radiation even using low dose protocols, and vascular studies require intravenous injection of iodinated contrast agents^6,13^.

MRI is widely used in anatomy and function assessment of CHD due to the absence of ionizing radiation^6,8^. The signal void of blood flow in SE sequences provides a good contrast between the vascular lumen and the vessel walls and excellent differentiation between surrounding soft tissues and mediastinal fat, providing additional diagnostic information. In the 1990s, the T1 weighted (T1W) SE sequence was the principal tool for the demonstration of tracheobronchial anomalies^5,7,13–16^. However, T1W SE with thin slices requires a long acquisition time and is sensitive to motion and 2D images produce suboptimal 3D multi-planar reconstructions; Hence, the three-dimensional anatomy of the tracheobronchial tree cannot be demonstrated adequately. In recent years, authors have noted that varying degrees of airway narrowing can be noted on 3D-bTFE images^17–19^. Then, by utilizing 3D-TFE, we detected 64% tracheobronchial anomalies in our group (41% tracheobronchial stenosis, 12% tracheal bronchus, 5% isomerism type of bronchus, 4% bridging bronchus, 2% situs inversus type of bronchus). 3D-TFE provided improved image quality as compared to that of 3D-bTFE. All patients underwent MSCT before or after MRI examination and there was a high inter-modality agreement between MSCT and 3D-TFE (*Kappa* = *0*.*75*). The sensitivity, specificity and accuracy of 3D-TFE(91.3%, 82.8%, 88.0%) were higher than 3D-bTFE(84.0%, 81.0%, 83.1%). The higher NPV and lower NLR of 3D-TFE(85.7%, 0.1) indicate that 3D-TFE sequence can demonstrate normal tracheobronchial pattern more correct than 3D-bTFE sequence. Although the PPV of 3D-TFE(89.0%) was lower than 3D-bTFE(91.3%), the difference between them is not obvious.

3D-TFE is a 3D fast gradient echo sequence that employs an inversion recovery radiofrequency pulse for magnetization preparation. It is designed for rapid acquisition with T1 weighting dominance. Fast gradient echoes are characterized by their rapid sampling time and high signal intensity and image contrast when approaching steady state. The rapid speed of acquisition makes 3D-TFE an excellent alternative cardiac imaging modality. Without fat suppression and T2 prep pulses in 3D-TFE sequence makes further enhance the contrast between the tracheobronchial tree and surrounding tissues compared with 3D-bTFE^19^. Navigator gating and ECG triggering are enabled to reduce artifacts from respiratory and cardiac motion. In MinIP reconstruction, the tracheobronchial tree can be visualized optimally. Performing 3D-TFE after contrast injection also enhances the contrast between the tracheobronchial tree and surrounding tissues. The relationship between the tracheobronchus and its surrounding vascular structures can be demonstrated through MIP reconstruction. 3D images overcome the disadvantages of axial images, such as the inability to identify focal narrowing, reliably estimate the longitudinal extent of disease and display complex 3D anatomic relationships.

3D-bTFE also can demonstrate tracheobronchial anomalies but the image quality is less optimal. 3D-bTFE has higher sensitivity to B0 field inhomogeneity and signal loss artifacts can be present at air-tissue interface and at the periphery of the field-of-view; thus, the trachea and bronchus, especially upper portion of trachea are not clearly observed^19^.

In this study, predominant causes of tracheobronchial stenosis were vascular abnormalities which included double aortic arch, right aortic arch with mirror-image branching, right aortic arch with aberrant left subclavian artery or posterior PDA or ligament, left aortic arch with right descending aorta, innominate artery compression syndrome, left pulmonary artery sling, absent pulmonary artery valve, enlarged left atrium and dilated ascending aorta. MRI can also be used to evaluate the other associated cardiac defects and cardiac function through cine MRI and phase contrast sequences.

There are some limitations in our study. First, there is a potential selection bias to this retrospective study and the expected frequency of these tracheobronchial anomalies in our group was higher. Second limitation is that there was no surgical/pathologic results to compare, but CT can be a good standard reference because of its advantage in demonstrating of lung and tracheobronchial tree.

## Conclusions

3D-TFE is a useful MRI sequence for the demonstration of the tracheobronchial tree and the diagnosis of tracheobronchial anomalies in congenital heart disease. MRI can supply helpful tracheobronchial information for preoperative strategies and postoperative follow-up.

## Methods

### Subjects

This retrospective research study was compliant with the Health Insurance Portability and Accountability Act and was approved by our tertiary care institutional review board. From December 1, 2013 to June, 2016, 75 patients with CHD (45 boys and 30 girls) who underwent both cardiac MRI and MSCT cardiac/chest examinations were enrolled. Their parents have signed informed consent before all the examination began. Patient ages ranged from 1.4 months to 134 months and the median age was 10.4 months. Cardiac MRI was performed to provide preoperative information about cardiovascular anatomy or/and cardiac function. MSCT was also performed either before or after the cardiac MRI examination. The time between the MSCT and cardiac MRI examination was 3.77 ± 7.08 months. MSCT examinations include ECG-gated cardiac CT (CCT), non-ECG-gated CT angiography (CTA) with intravenous contrast or CT of the chest (chest CT) without contrast.

### MRI and CT examination

All MRI examinations were performed on a 1.5 T clinical MRI system (Achieva Philips Healthcare, Best, The Netherlands) with a 16-channels torso or 8-channels cardiac phased-array coil. Two-dimensional cine balanced turbo field echo (2D cine b TFE), ECG-triggered 3D-bTFE, 3D-TFE with respiratory navigator, and contrast enhanced magnetic resonance angiography (CE-MRA) were performed. 2D cine bTFE, 3D-bTFE and CE-MRA were performed to evaluation cardiovascular anomalies and cardiac function. The ECG-triggered 3D-TFE sequence with respiratory navigator was performed on patients to evaluate the tracheobronchial tree. The acquisition parameters of these 4 sequences were summarized in Table 3.

**Table 3:** The acquisition parameters in four MR sequences.

	2D-cine bTFE	3D-bTFE	3D-TFE	CE-MRA
TR/TE(ms)	3.5/1.7	4.7/2.4	5.6/1.7	2.0/1.1
Flip Angle (degrees)	60	90	30	35
FOV	220–300	260–400	260–400	260–400
Number of slice	12–16	70–90	70–90	70–90
Acquired voxel sizes (mm^3^)	1.3 × 1.6 × 6–8	1.0–1.3 × 1.0–1.3 × 1.0–1.3	1.0–1.3 × 1.0–1.3 × 1.0–1.3	1.3 × 1.3 × 1.8
Reconstructed voxel sizes (mm^3^)	1.0 × 0.9 × 6–8	0.57 × 0.57 × 1.0–1.3	0.57 × 0.57 × 1.0–1.3	0.76 × 0.76 × 0.9
SENSE acceleration factor	2.0	2.0	2.0	1.8
number of signal acquisitions	3–4	1	1	1
Navigator gating	No	yes	yes	No
ECG triggering	yes	yes	yes	No

TR, repetition time; TE: echo time; FOV: field of view.

All patients also underwent either low dose chest CT or CCT, or CTA before or after MRI examination. MSCT with 16 slices (General Electric Medical Systems, Milwaukee, WI, USA) or 64-slice high definition CT scanner (Discovery HD 750, GE Healthcare, Waukesha, WI, USA).

The parameters for CCT using the 64-slice CT scanner with prospective scanning mode was as follows: step-and-shoot axial scanning and a collimation of 64 × 0.625 mm with a scan field of view of 25 cm and gantry rotation time of 0.35 s; Tube voltage of 80–100 kV and tube current of 35–70 mA. All scans were reconstructed using 80% ASIR (adaptive statistical iterative reconstruction) algorithm.

The parameters for CTA using 16 slice MDCT with non-ECG triggering scanning mode were as follows: 120 kV, 100–200 mA, 0.625 collimation 5.62 mm/s table speed, rotation speed 0.5 and 0.3-mm reconstruction.

To decrease contrast medium-related artifacts and to achieve homogeneous contrast enhancement, MSCT was performed in the caudocranial direction when an arm vein was used and craniocaudal direction when a lower extremity vein was used. The injection rate using a power injector was 0.8–2.5 ml/s according to the scan range. The scan delay was 13–18 s depending on the site of injection or using bolus tracking. Non-ionic contrast agent (2 ml/kg), Iopamidol 370 mg/ml (Bracco, Milan, Italy), was administered.

The parameters for chest CT on 16-slice CT scanner were as follows: 120 kV, 50 mA, 7.5 mm, Collimation, pitch 1.375: 1, and 1.25 mm reconstruction.

### Image Processing and Imaging Quality Evaluation

The 3D datasets of MRI and CT were processed on an offline EWS workstation (Philips Healthcare, Best, The Netherlands), and Advantage Windows workstation 4.2–4.6 (General Electric Medical Systems, Milwaukee, USA) with maximum intensity projection (MIP) and minimum intensity projection (MinIP) algorithms and volume render techniques.

The anatomy of the tracheobronchial tree and its anomalies in 75 patients were blindly evaluated by two pediatric radiologists with 14-years (Q. Wang) and 13-years experiences (A.M. Sun) in thoracic imaging, and the reviewing process was consensus reading by these two reviewers. We defined the local or long segment tracheobronchial stenosis and did not define the severity of stenosis (for instance: mild, moderate and severe stenosis). More than one-third of the tracheal length is called long-segment stenosis; otherwise it is local or short-segment stenosis^20^. The radiologists also independently evaluated image quality of 3D-TFE and 3D-bTFE based on reconstructed MinIP images on a five-point scoring system: 5 = excellent anatomical clarity and image quality (clear visualization of tracheobronchial tree margin and two generations of bronchus); 4 = good anatomical clarity and image quality (minor respiratory motion artifact); 3 = fair image quality (tracheobronchial tree margin is not very clear but still visualized); 2 = poor image quality (inadequate delineation between the airway and the surrounding tissue), and 1 = non-diagnostic image quality. Scored over 3 were considered sufficient for diagnosis.

### Statistical analysis

Quantitative data were expressed as means ± standard deviations, and categorical data were given in proportions and percentages. The inter-modality agreement for tracheobronchial anomaly findings was tested by the *kappa* coefficient. A value of 0.8 or above indicates excellent agreement, 0.6–0.8 good agreement, 0.4–0.6, fair agreement and <0.4 poor agreement. Comparing the CT findings, the sensitivity, specificity, PPV, NPV, PLR and NLR of 3D-TFE and 3D-bTFE for the detection of tracheobronchial anomalies were evaluated for each observer using the consensus reading of the MSCT findings as the standard reference. Statistical analysis was performed using SPSS version 19.0 (IBM, Armonk, NY, USA). A *P* value < 0.01 indicated statistical significance.

### Availability of data and materials

The data that support the findings of this study are available from corresponding author upon reasonable request.

### Ethics approval and consent to participate

This study was approved by the review board of Shanghai Children’s Medical Center Ethics Committee. ID nr: SCMCIRB-K2016009. This retrospective research study was compliant with the Health Insurance Portability and Accountability Act and all patients and/or guardians have signed informed consent before all the examination began.

## Electronic supplementary material

Supplementary information
