# Supplementary material for: Diagnostic Accuracy of Three-dimensional Turbo Field Echo Magnetic Resonance Imaging Sequence in Pediatric Tracheobronchial Anomalies with Congenital Heart Disease
Source: Sci Rep. 2018 Feb 7;8:2529. doi: 10.1038/s41598-018-20892-2 (PMC5803237; doi:10.1038/s41598-018-20892-2)
Supplement: Supplementary file 1 — Supplementary information [file 41598_2018_20892_MOESM1_ESM.doc]

**Title page**

**•Title**

Diagnostic Accuracy of Three-dimensional Turbo Field Echo Magnetic Resonance Imaging Sequence in Pediatric Tracheobronchial Anomalies with Congenital Heart Disease

**•Type of Manuscript**

Full Paper

**•Authors information**

***Note***: QiaoRu Hou and Wei Gao have equal contribution for this manuscript, so they are both first author (equal contributors, co-author).

1. QiaoRu Hou, Diagnostic imaging Center of Shanghai Children’s Medical Center affiliated with Shanghai Jiao Tong University Medical School, Shanghai; No.1678 Dong Fang Road, Shanghai 200127 P.R.China houqiaoru@163.com
2. Wei Gao, Department of Pediatric Cardiology of Shanghai Children’s Medical Center affiliated with Shanghai Jiao Tong University Medical School; No.1678 Dong Fang Road, Shanghai 200127 P.R.China davidgao1963@163.com
3. YuMin Zhong, Diagnostic imaging Center of Shanghai Children’s Medical Center affiliated with Shanghai Jiao Tong University Medical School; No1678 Dong Fang Road, Shanghai 200127 P.R.China zyumin2002@gmail.com
4. AiMin Sun, Diagnostic imaging Center of Shanghai Children’s Medical Center affiliated with Shanghai Jiao Tong University Medical School, Shanghai; No1678 Dong Fang Road, Shanghai 200127 P.R.China aiminsun217@yahoo.ca
5. Qian Wang, Diagnostic imaging Center of Shanghai Children’s Medical Center affiliated with Shanghai Jiao Tong University Medical School, Shanghai; No1678 Dong Fang Road, Shanghai 200127 P.R.China wangqiak@hotmail.com
6. LiWei Hu, Diagnostic imaging Center of Shanghai Children’s Medical Center affiliated with Shanghai Jiao Tong University Medical School, Shanghai; No1678 Dong Fang Road, Shanghai 200127 P.R.China huliwei11@hotmail.com
7. JingLei Wang, Diagnostic imaging Center of Shanghai Children’s Medical Center affiliated with Shanghai Jiao Tong University Medical School, Shanghai; No1678 Dong Fang Road, Shanghai 200127 P.R.China wangjinglei@scmc.com.cn

Corresponding author: YuMin Zhong

Address: No1678 Dong Fang Road Shanghai

E-mail: zyumin2002@gmail.com

Tel: 8621-38626161-5001

Fax:8621-58393915

**Supplementary Tables**

Supplementary Table S1

Clinical information

| CHD | Cyanotic CHD | Acyanotic CHD |
| --- | --- | --- |
| Tetralogy of Fallot | 13 |  |
| pulmonary atresia with ventricular septal defect | 4 |  |
| double outlet right ventricle | 2 |  |
| corrected transposition of great arteries | 1 |  |
| truncus arteriosus | 1 |  |
| single ventricle | 3 |  |
| total anomalous pulmonary venous connection | 1 |  |
| ventricular septal defect and atrial septal defect |  | 32 |
| patent ductus arteriosus |  | 2 |
| pulmonary stenosis |  | 3 |
| aortic stenosis |  | 1 |
| coarctation of aorta |  | 2 |
| atrioventricular septal defect |  | 1 |
| supravalvar stenosis |  | 2 |
| Left pulmonary artery sling |  | 1 |
| absent right pulmonary artery |  | 1 |
| Absent pulmonary artery valve |  | 1 |
| PAPVC |  | 1 |
| Double aortic arch with or without VSD or ASD |  | 3 |
| Total | 25 | 50 |

Supplementary Table S2

Validity and efficacy of 3D-TFE sequence in the diagnosis of tracheobronchial anomalies in 75 patients

| MRI  MSCT | Tracheobronchial anomaly | Normal tracheobronchial tree | Total |
| --- | --- | --- | --- |
| Tracheobronchial anomaly | 42 | 5 | 47 |
| Normal tracheobronchial tree | 4 | 24 | 28 |
| Total | 46 | 29 | 75 |

Supplementary Table S3

Validity and efficacy of 3D-bTFE sequence in the diagnosis of tracheobronchial anomalies in 71 patients

| MRI  MSCT | Tracheobronchial anomaly | Normal tracheobronchial tree | Total |
| --- | --- | --- | --- |
| Tracheobronchial anomaly | 42 | 4 | 46 |
| Normal tracheobronchial tree | 8 | 17 | 25 |
| Total | 50 | 21 | 71 |
